# NMDA receptor modulation and severe acute respiratory syndrome treatment

**DOI:** 10.12688/f1000research.73897.1

**Published:** 2021-10-18

**Authors:** Blaise M. Costa

**Affiliations:** 1 Center for One Health Research, Virginia Polytechnic Institute and State University, Blacksburg, VA, 24060, USA

**Keywords:** NMDA, severe respiratory syndrome (SARS), GluN2D, Potentiator

## Abstract

N-Methyl-D-aspartate (NMDA) subtype of glutamate receptors is expressed in the human lungs and central nervous system.  NMDA receptor potentiation could increase calcium ion influx and promote downstream signaling mechanisms associated with cellular contractions that are disrupted in severe acute respiratory syndrome. Pharmacological effects generated by triggering glutamate receptor function in the brain, coupled with concurrent stimulation of the respiratory tract, may produce a synergetic effect, improving the airway smooth muscle function. A novel multipronged intervention to simultaneously potentiate NMDA receptors expressed both in the central nervous system and airway muscles would be helpful for the treatment of severe acute respiratory syndrome that deteriorates peripheral and central nervous system function before causing death in humans.

Glutamate is the major neurotransmitter of the central nervous system and it has diverse roles in the periphery. The N-methyl-D-aspartate (NMDA) receptor is a major subtype of glutamate receptors, which are predominantly expressed throughout the nervous system and in all vital organs in the human body. ^1^ Functional NMDA receptors are hetero-tetramers composed of two identical glycine binding GluN1 subunits and two identical or different glutamate binding GluN2 subunits, of which there are four subtypes, GluN2A-D. ^2^^,^^3^

## Expression of NMDA receptors in the human lungs

The Human Proteome Project identified abundant expression of NMDA receptor subunits in various organs outside the CNS, including lungs, esophagus, and T-helper cells. ^1^ This finding corroborates a large number of previous reports on the extraneuronal expression of NMDA receptors in various animals. ^1^^,^^4^^–^^12^ NMDA, when applied to perfused tracheal segments of guinea pigs, increased resting muscle tone and enhanced the contractile response to acetylcholine. ^13^^,^^14^ In whole guinea pig lungs, when administered through the trachea, NMDA increased airway perfusion pressure and this increase was abolished by NMDA receptor channel blocker MK-801(4). Following systemic MK-801 administration, adult cats developed apneusis. ^15^^,^^16^ In addition, recent studies reveal the critical role of endogenous glutamate in NMDA receptor function during acute lung injury and airway inflammation. ^4^^,^^14^^,^^17^^,^^18^ An NMDA receptor blocker could impair fetal rat lung development. ^19^^,^^20^ NMDA receptor activation mediates lung fibroblast proliferation and differentiation in hyperoxia-induced chronic lung disease in newborn rats. ^20^ Acute lung injury, acute respiratory distress syndrome and severe acute respiratory syndrome (SARS) all imply the occurrence of lung injury resulting from direct or indirect respiratory insult. ^21^

The expression of GluN1/2A and 1/2B subtypes were not confirmed in the lung cells, whereas the GluN1/2C subtype was found to be expressed in peripheral and middle-lobe lung samples. ^4^ The GluN1/2D subunit was predominantly expressed in the peripheral, gas-exchange zone of the lungs and in alveolar macrophages; this expression was upregulated in lungs treated with NMDA. ^4^ GluN1 and all four GluN2 subunits were also expressed in the human pulmonary artery smooth muscle cells. ^22^ Overall, these findings indicate that NMDA receptors could control the respiratory tract function in vertebrate animals. ^23^

## Kinetics of NMDA receptor subtypes

Glutamate, with concurrent binding of the co-agonist D-serine or glycine, activates NMDA receptors that non-selectively conduct ions across the cells at depolarizing membrane potential which unbinds the otherwise blocking Mg ^2+^ ions. NMDA receptor mediated transport of calcium and sodium ions into the cytoplasm is essential for excitatory cellular events that result in human airway smooth muscle contraction. ^24^ Each non-GluN1 subunit confers distinct spatiotemporal expression and biophysical properties that result in varying agonist affinity, magnesium sensitivity, ion conductance, activation kinetics, open probability, mean open time, cellular localization, and downstream signaling mechanisms. ^2^ In general, diheteromeric NMDA receptors (GluN1/2) exhibit deactivation time constants that span about a 50-fold range, with the following order (from fastest to slowest): NR2A < 2C < 2B << 2D. ^25^ The GluN1/2A subunit-containing NMDA receptor deactivation time constant is about ~50 ms, GluN1/2B ~400 ms, GluN1/2C ~290 ms and GluN1/2D is >1second. ^25^ Since GluN1/2C&D subunits of NMDA receptors are predominantly expressed in the lung epithelial cells and macrophages, and are the slowest channel (among other glutamate receptors) to deactivate, these receptors can conduct a large amount of calcium and sodium ions into the cells and trigger cellular contractions. ^25^^–^^27^

## Antiviral properties of drugs acting on NMDA receptors

One of the clinically used antiviral agents, amantadine, is a potent NMDA receptor antagonist. ^28^ This drug is also an FDA-approved drug of choice (brand name, Gocovri ^®^) for the treatment of dyskinesia in patients with Parkinson’s disease. An analog of amantadine, memantine (brand name, Namenda ^®^), is one of two FDA-approved clinically used drugs for the treatment of moderate to severe symptoms of Alzheimer’s disease. Since both amantadine and memantine are chemically similar adamantane derivatives, memantine also exerts antiviral effects as previously reported. ^29^ Presumably, these effects could be a collective outcome of activities on host cell glutamate receptors and viral proteins like M2-viroporin. ^30^

## Novel NMDA receptor modulators

In recent years, a variety of NMDA receptor modulators have been identified, and they exhibit a broad spectrum of subunit selectivity and mechanisms of action. ^31^^–^^35^ These compounds have been largely studied for their activities in neuronal NMDA receptor populations, with the aim of developing treatments for neurological and psychiatric disorders; however, these compounds and their analogs might have therapeutic potential for non-CNS disorders, but this has not yet been explored.

Through our ongoing NMDA receptor drug discovery project, we have identified a compound from PubChem (CID# 3794169), coded as CNS4, and studied its activity on NMDA receptors. ^36^ CNS4 selectively potentiates GluN1/2D receptor currents up to 8-fold, when activated by 100 μM glycine and 0.3μM glutamate, and produces minimal effects on GluN1/2A or 1/2B receptors. ^36^ CNS4 has a variety of other biological activities as reported by the National Center for Advancing Translational Sciences ( NCATS); for example: an inconclusive anti-viral activity against influenza-A virus non-structural protein-1 (PubChem AID# 2326); anti-malarial, as an inhibitor of apical membrane antigen-1 of *Plasmodium falciparum* (AID# 720542); antiprotozoal, as an inhibitor of fructose 1,6- bisphosphate aldolase from *Giardia* Lamblia (AID# *lamblia* (2451); and inhibition of nuclear receptor ROR-gamma in the immune cells (AID# 2551 & 2546). The chemical structure of CNS4 and more details on its activities are available at PubChem.

## A multipronged approach to treat SARS

An NMDA receptor modulator with antiviral properties could serve as a novel treatment strategy for SARS. Potentiating NMDA receptor activity in the lung epithelial cells will increase calcium ion influx and promote downstream signaling mechanisms associated with cellular contractions that are possibly impaired during SARS. Pharmacological effects generated by triggering neuronal NMDA receptor function, coupled with concurrent potentiation of NMDA receptors expressed in the respiratory tract, could synergistically improve airway smooth muscle contractions. Further, a variety of neurological symptoms were clinically diagnosed in hospitalized COVID-19 patients. ^37^^,^^38^ Neuropathogenesis could occur due to the neurologic injury resulting from systemic dysfunction, ^39^ dysregulated renin-angiotensin aldosterone system, ^40^ proinflammatory reactions, ^41^^,^^42^ para-infectious and post-infectious triggers, ^43^ and direct viral invasion of the nervous system. ^44^^–^^46^ As a well characterized neuropsychiatric drug target, ^47^ with the potential to improve lung function, NMDA receptors could be an ideal focal point for future pharmacological interventions of COVID-19. Clinical conditions involving hypoxia increase blood glutamate concentration by promoting transaminase activity that generates α-keto acids. ^48^^–^^50^ Further, neuronal glutamate excitotoxicity induces paralysis in mice after infection by a human coronavirus. ^51^ The connection between disruption in glutamate homeostasis and pathogenesis of various neurological and psychiatric disorders has been extensively studied in the past three decades. Therefore, optimizing glutamatergic signal transmission through neuronal and non-neuronal cell types, that express the major glutamate receptor subtypes like NMDA receptor, could be an appropriate strategy to reduce the extent of lung damage caused by SARS. In this perspective, compounds that modulate NMDA receptors based on glutamate concentration would be an ideal starting point for the development of a treatment approach involving modulation of glutamate signaling at both nerve cells and non-neuronal cells. CNS4 and other recently identified novel glutamate concentration biased NMDA receptor modulators ^34^^,^^35^ could serve as lead candidates in the development of clinically useful compounds to treat COVID-19 or other SARS caused by various pathological conditions. Future studies should be carried out in this direction to test this hypothesis.

## Conclusion

An increasing body of evidence suggests the expression of functional NMDA receptors in the lungs and their critical role in glutamate induced acute lung injury and acute respiratory distress syndrome. ^4^^,^^13^^,^^14^^,^^17^^–^^20^ Despite its direct role in lung injury, little effort has been taken to develop NMDA receptor based therapeutic strategies for the treatment of lung diseases. With the revolution in glutamate receptor pharmacology in the past decade that yielded a variety of chemical tools to modulate NMDA receptors, ^31^^–^^36^ and a COVID-19 pandemic that kills humans by primarily affecting lung function, this could be a suitable time to start working on a novel drug target for SARS treatment.

## Data availability

No data is associated with this article.
