# Supplementary material for: NMDA receptor modulation and severe acute respiratory syndrome treatment
Source: F1000Res. 2021 Oct 18;10:Chem Inf Sci-1060. [Version 1] doi: 10.12688/f1000research.73897.1 (PMC9745209; doi:10.12688/f1000research.73897.1)
Supplement: Peer review history

# Peer review: Reviewer response for version 1

**DOI:** 10.5256/f1000research.77586.r157840

**Published:** 2022-12-12

**Authors:** Jaewon Ko

**Affiliations:** 1 Daegu Gyeongbuk Institute of Science and Technology (DGIST), Daegu, South Korea

This short article states that modulation of an NMDA receptor (NMDAR) function with anti-viral properties could be beneficial for treating disorders that involve NMDAR dysfunctions. The author first summarized known facts for distinct NMDAR subunits expressed in lung cells (i.e., expression levels and kinetics). The author then introduced amantadine (a potent NMDAR antagonist) and its analog (memantine) that have been clinically used for treatment of dyskinesia patients. The author also detailed a novel NMDAR modulator (i.e., CNS4) that has a possibly therapeutic potential for non-CNS disorders and SARS.

 I believe that the author has done a wonderful job in concisely providing a logical opinion: a newly developed NMDAR modulator could be clinically promising for treating various non-neurological disorders that are linked to NMDAR dysfunctions.

Is the topic of the opinion article discussed accurately in the context of the current literature?

Yes

Are arguments sufficiently supported by evidence from the published literature?

Yes

Are all factual statements correct and adequately supported by citations?

Yes

Are the conclusions drawn balanced and justified on the basis of the presented arguments?

Yes

Reviewer Expertise:

Synaptic neuroscience.

I confirm that I have read this submission and believe that I have an appropriate level of expertise to confirm that it is of an acceptable scientific standard.

# Peer review: Reviewer response for version 1

**DOI:** 10.5256/f1000research.77586.r142691

**Published:** 2022-07-25

**Authors:** James Pearle

**Affiliations:** 1 California Research Medical Group, Fullerton, USA

In severe acute respiratory distress syndrome, cellular contractions may be disrupted. 

 By triggering glutamate receptor function in the brain and with stimulation of the respiratory tract, a synergetic effect may improve airways smooth muscle function and neurologic function.

 Stimulation of NMDA receptors in the brain and in the airway muscles could be beneficial in the treatment of severe respiratory distress syndrome and associated neurologic disease.

 The authors excellently outline how an NMDA receptor modulator with antiviral properties could be a novel treatment strategy for SARS.

 This avenue of treatment has the potential to prevent or treat the neurological dysfunction often associated with SARS.

 At the same time, NMDA receptor modulation may improve pulmonary status by improving smooth muscle airways function, often disrupted in this syndrome.

 The multifaceted potential benefits of stimulating glutamate receptors could involve both the lungs and the nervous system.

 The authors have clearly and concisely outlined the rationale and hypothesis for the use of NMDA modulators in the treatments of SARS and associated neurologic dysfunction.

Is the topic of the opinion article discussed accurately in the context of the current literature?

Yes

Are arguments sufficiently supported by evidence from the published literature?

Yes

Are all factual statements correct and adequately supported by citations?

Yes

Are the conclusions drawn balanced and justified on the basis of the presented arguments?

Yes

Reviewer Expertise:

Pulmonologist

I confirm that I have read this submission and believe that I have an appropriate level of expertise to confirm that it is of an acceptable scientific standard.
